# Study of Relationships between Ceiling Smoke Leakage Rate and Evacuation Time in the Ward

**DOI:** 10.3390/ijerph182413280

**Published:** 2021-12-16

**Authors:** Shuo-Hong Liu, Ching-Yuan Lin, Ying-Ji Chuang

**Affiliations:** Department of Architecture, National Taiwan University of Science and Technology, Taipei City 10607, Taiwan; linyuan@mail.ntust.edu.tw (C.-Y.L.); d9413005@gmail.com (Y.-J.C.)

**Keywords:** ward, evacuation time, ceiling, smoke, leakage

## Abstract

With reference to the requirements of CNS 15038 and testing principles, this study proposes a set of equipment for measuring the leakage volume of ceilings and provides detailed assembly specifications for future users. In this study, a total of 405 tests were conducted as part of a set of experiments for measuring the leakage volume of ceilings, using various ceiling materials, ceiling sizes, and construction methods, in conjunction with the principles of fluid mechanics, to propose a method for evaluating the leakage volume of ceilings of various sizes and materials. Two cases—bottom-up airflow and top-down airflow—were considered. According to our research findings, in the case of bottom-up airflow, the pressure difference, panel weight, and panel size were correlated with the leakage volume; the more significant the pressure difference, the larger the leakage volume; the heavier the panel weight, the more minor the leakage volume; and the larger the panel size, the more significant the leakage volume. On the other hand, in the case of top-down airflow, different leakage volumes were observed for different ceiling materials, even if the ceiling size was identical. On the other hand, when the ceiling material was the same, and the ceiling size was different, there was not a positive relationship between the leakage volume and a larger panel size; instead, the leakage volume observed for the largest panel was the smallest. Finally, in this study we propose a volumetric leakage assessment table for assessing a ceiling as a whole, which can be utilized by engineers in the future to calculate the smoke leakage value and to estimate the smoke fall time for ward escape designs.

## 1. Introduction

**Figure 1:**
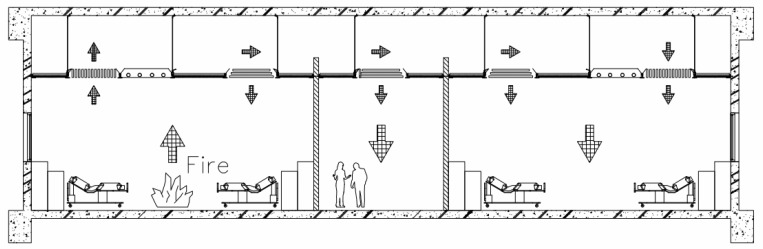
Schematic diagram of the smoke leakage path from the ceiling.

The smoke from a building fire contains dangerous gases such as carbon monoxide (CO), hydrocyanic acid (HCN), ammonia (NH_3_), sulfur dioxide (SO_2_), and hydrochloric acid (HCl), among which the most severe and common gas is carbon monoxide. Previous studies have confirmed that carbon monoxide is the leading cause of most cases of death by poisoning [1], and people with cardiovascular or respiratory diseases are particularly vulnerable to carbon monoxide. The inhalation of carbon monoxide will rapidly reduce the oxygen concentration in the blood and trigger life-threatening effects. The smoke generated by burning objects during a fire is very voluminous. If the overall airtightness of a building is increased, the smoke cannot be exhausted to the outside of the building and may lead to a deterioration in the safety environment for evacuees and rescuers during a fire [2]. Smoke damage has been often reported in previous fire case studies to cause loss of life earlier than the temperature of the fire [3]. As the temperature rises, gases expand rapidly in a fire, creating pressure on floors, floors, and walls, with smoke passing from the high-pressure sides to the low-pressure sides [4]. In terms of living room fires, the smoke flow can be divided into three paths: first, the spread through holes and openings, second, the spread through pipes and crevices in the walls, and third, the spread through ceilings. Smoke flow behaviors are very complex in buildings and have been studied previously [5,6,7,8] in various theoretical analyses. The proposed solutions for this problem are straightforward, such as confining the smoke generated by combustion to a particular area; however, the interiors of buildings contain many pipes through the ceiling, floor, and walls, as well as doors and windows in the walls and other objects, so these gaps [9] and openings need to be covered, and any equipment [10] needs to be covered. Recently, there have been many incidents of fires in nursing homes in Taiwan. As most of the residents in these wards are elderly, immobile, or even vulnerable, depending on the presence of life-supporting systems, it is not easy for nursing staff members, who are often few in number, to handle the large-scale evacuation of the residents, nor is it possible to use the stairs for a vertical evacuation [11,12]. Therefore, immediate fire-fighting efforts are required [13], with the hope of extending the response period by means of fire and smoke protection zones so that medical life-support systems can continue to operate [14]. Doors and walls of wards need to be fire-resistant as an essential requirement, and doors must also have smoke-shielding properties, which is currently regulated by various countries [15,16,17,18,19,20,21,22,23]. However, there may be maintenance pipes running between wards, and the walls between wards are sometimes not connected to the underside of the floor (Figure 1), and hospitals are most often equipped with suspended ceiling systems. Therefore, when a fire breaks out in one ward, smoke may be transmitted through the gap in the ceiling to the interior of the ceiling, with the smoke then traveling down to another ward where there is no fire. This aspect is often neglected, although there have been studies on the calculation of smoke movement through air supply and exhaust systems [24] and a study by Chou et al. [25] on the basic leakage volume of ceilings. However, the relationship between the leakage volume of smoke from various ceiling materials and the evacuation time has not been studied for various pressure differential environments. This study aims to propose the application of evacuation time parameters for suspended light-steel-frame ceilings in ward design through field leakage volume tests and theoretical analysis [26].

## 2. Experimental Description

### 2.1. Experimental Design

**Figure 2:**
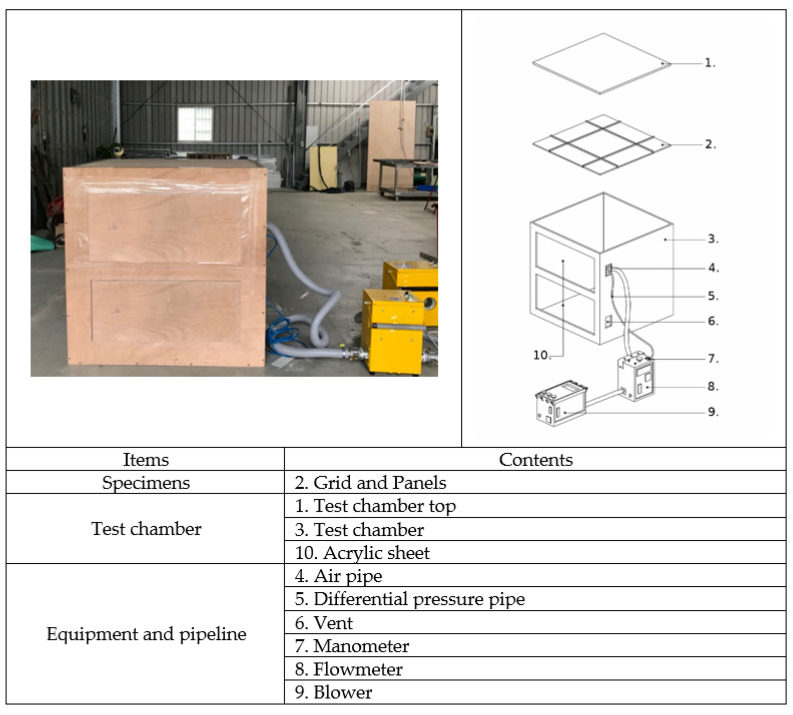
Test equipment composition.

**Figure 3:**
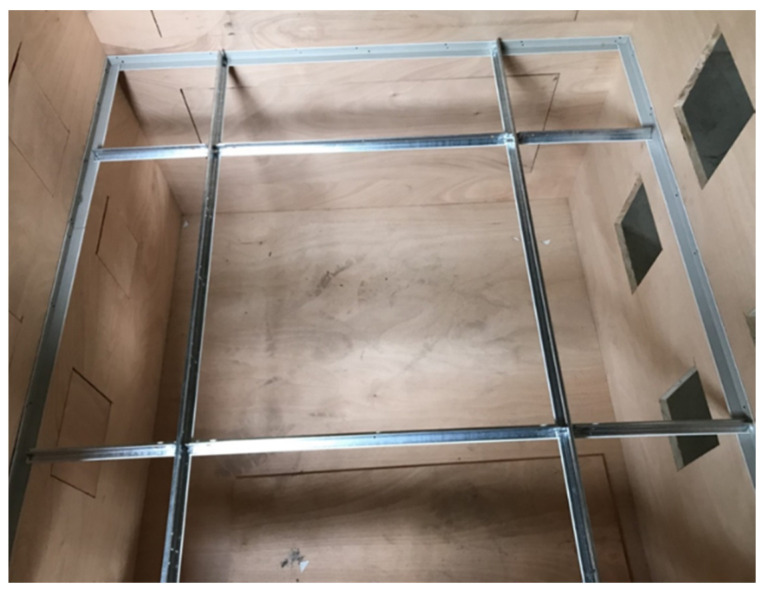
Installation of open-frame ceiling main frame and subframe in the test chamber.

**Figure 4:**
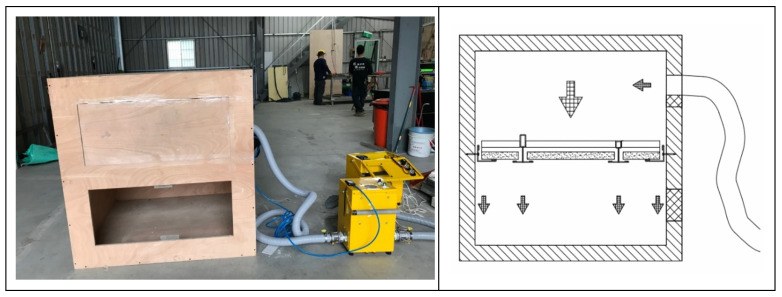
Schematic diagram of the smoke leakage path from the top to the bottom of the equipment in the test chamber.

**Figure 5:**
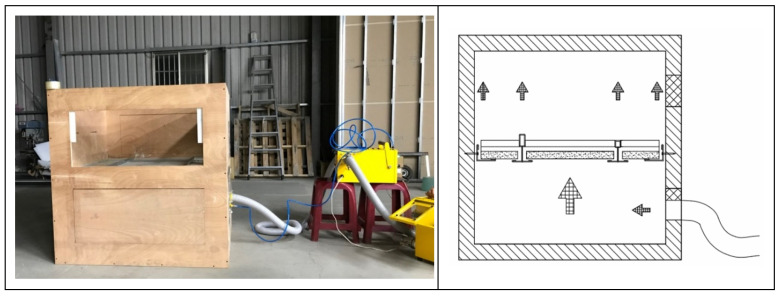
Schematic diagram of the smoke leakage path from the bottom to the top of the equipment in the test chamber.

As shown in Figure 2, the structure of the test chamber is formed by a wooden board with a thickness of 24 mm and a density of 0.353 g/cm^3^ and is equipped with a transparent acrylic observation port for observing the changes in the ceiling specimen; as shown in Figure 3, the internal dimensions of the test chamber are 1.225 m (height) × 1.225 m (width) × 1.225 m (depth); the wooden boards on the inner side of the test chamber surface seams are covered with airtight tape. The wooden boards are connected with strong adhesives to ensure the overall airtightness of the test chamber. The test chamber is equipped with a 6 mm round hole on the side for metering of the differential pressure (pressure difference), which can measure 0–100 hPa with an accuracy of ±0.03 hPa, as well as an air blower (blower) and a flow meter (volume flow meter). The maximum air blower capacity is 6.8 m^3^/min, 1/4 HP, with 220 V voltage, three-phase electricity, and an inverter to control the blower speed (with a frequency control range of 0.01–650.00 HZ). The inner diameter of the air outlet is 50 mm, and this outlet is connected to the round hole in the test chamber. The flow meter can measure 0–75 m^3^/h with an accuracy of ±2.5% and can be used for fluid temperatures of −10 °C–60 °C and humidities of 90% or less, located between the blower outlet and the test chamber, with an inner diameter of 50 mm between the outlet and the inlet of the flow meter. In this study, a full-scale experimental design was carried out for simulation verification. The test chamber was equipped with a ventilation port on the non-pressurized side to facilitate leakage volume testing so that the air on the non-pressurized side and outside the test chamber can be balanced and no pressure difference is generated. When testing the smoke flow from the top to the bottom of the ceiling specimen, one connects the air duct of the blower and the positive pressure duct of the differential pressure meter to the upper position of the test chamber and the negative pressure duct of the differential pressure meter to the lower position of the test chamber, with a ventilation port on the non-pressurized side of the test chamber, as shown in Figure 4. When testing the bottom-up smoke flow behavior of the ceiling specimen, one connects the blower duct and the positive pressure duct of the differential pressure meter to the lower position of the test chamber and the negative pressure duct of the differential pressure meter to the upper position of the test chamber, with a ventilation port on the non-pressurized side of the test chamber, as shown in Figure 5. The measurement range of the relative humidity meter is 0%RH–100%RH, with a resolution of 0.1%RH. The measurement range of the thermometer is from −40 °C to +100 °C with a resolution of 0.1 °C. The measurement range of the atmospheric pressure meter is 300–1200 hPa, with a resolution of 0.1 hPa.

### 2.2. Experimental Specimens

**Figure 6:**
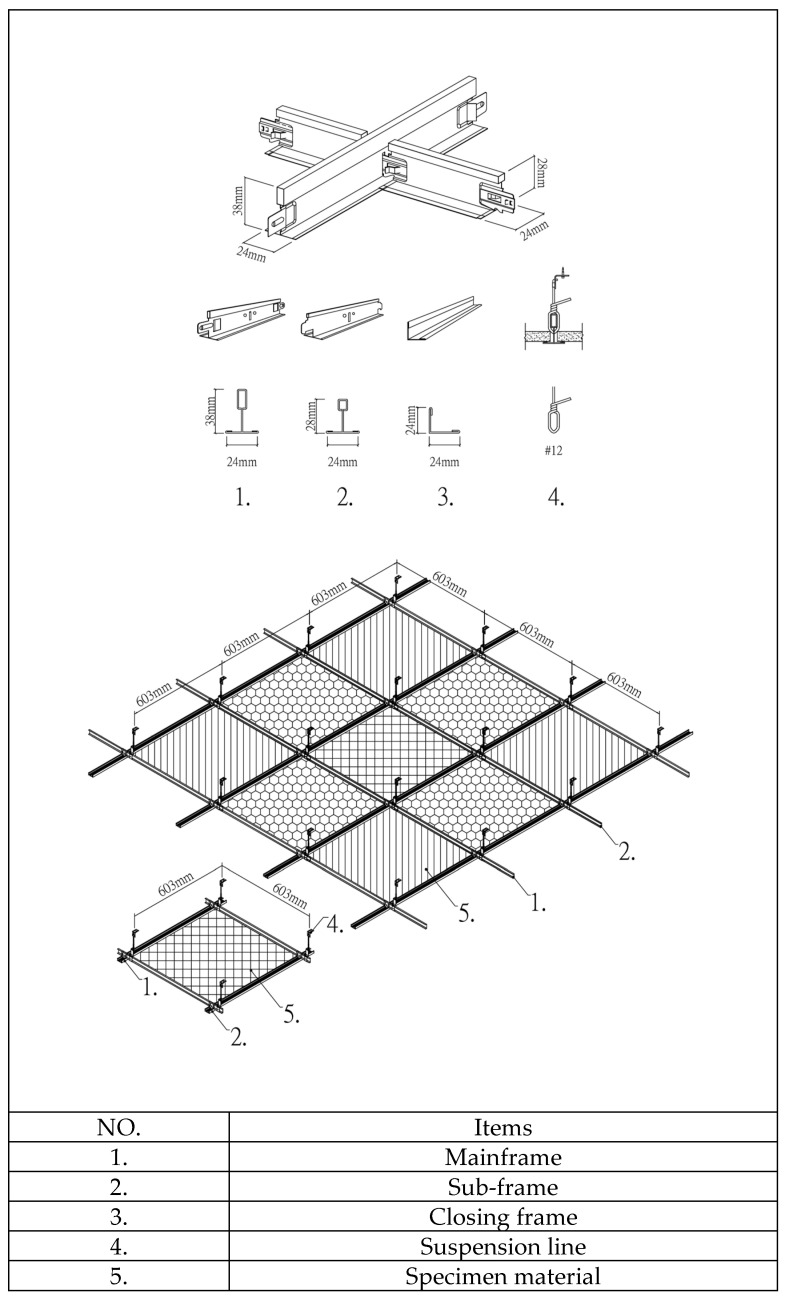
Suspension ceiling system.

**Figure 7:**
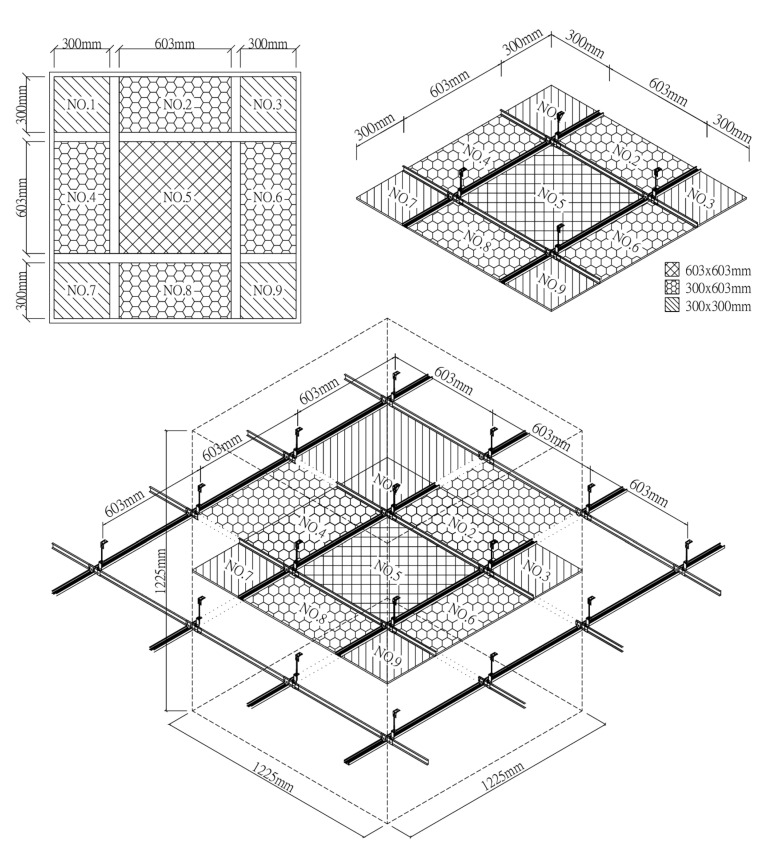
Test chamber ceiling number.

The suspended light-steel-frame ceiling system components include mainframes, subframes, hemming materials, suspension wires, and fixing screws (Figure 6), which are placed on the grid beam system formed by the connection of mainframes and subframes. Suspended light-steel-frame ceilings are often utilized in wards and can be used in earthquakes with a peak acceleration below 1600 gal [27]. In this study, the commonly used thicknesses of 9 mm gypsum board, 3.5 mm calcium silicate board, and 15 mm glass fiber board were used as samples, with densities of 0.75 g/cm^3^, 1.05 g/cm^3^, and 0.064 g/cm^3^, respectively, and bending damage loads of 36.3 kg/cm^2^, 13.1 kg/cm^2^, and 5.1 kg/cm^2^, respectively; in addition, conventional luminaires (type: 4 10 W T8 lamps, height: 52 mm, material: polished steel plate + white baking paint, weight: 3.13 kg), LED flat panel luminaires (power: 40 W, height: 11 mm, PC cover + white aluminum frame, weight: 2.02 kg), air return panels (type: pattern, material: aluminum, weight: 0.62 kg), and filter air return panels (type: pattern, material: aluminum, black foam laminated on the back). The test chamber ceilings were numbered from NO.1 to NO.9 (Figure 7) to obtain each ceiling specimen’s leakage volume. The plan dimensions of the panels, lights, and air return panels were 603 × 603 mm (2’ × 2’). Before testing of the suspended light-steel-frame ceiling system, samples were placed using a leveling tape according to the installation method at the construction site to make the ceiling system appear horizontal and increase the reliability of the test data.

### 2.3. Experimental Procedure

The experimental procedure was carried out with reference to the provisions of CNS 15038 [23]; each sample was tested five times to obtain its average leakage volume, primarily to measure the corrected leakage volume at 10 Pa, 25 Pa, and 50 Pa pressure differences (the measured leakage volume must be corrected to the leakage volume under standard conditions (temperature 20 °C (293.15 K) and one standard atmospheric pressure (101,325 Pa)). The correction calculation method (Equation (1)), as required by CNS 15038 [23], is as follows:

(1) $$Q_{a}’=\frac{Q_{a}}{\left(T+273.15\right)}\times \left[k\times (p_{a}+p_{m})-3.795\times 10^{-3}\times M_{w}\times p_{H_{2}O}\right]$$

$Q_{a}’$ is the actual leakage volume of the test specimen under the standard conditions of gas (m^3^/h); $Q_{a}$ is the actual leakage volume of the specimen at a temperature of (T+273.15) and pressure of $\left(p_{a}+p_{m}\right)$ (m^3^/h); $Q_{b}$ is the leakage volume of the chamber itself (m^3^/h); $Q_{t}$ is the leakage volume of the test specimen and the chamber itself (m^3^/h); k is a constant (293.15/10,1325) = 2.89 × 10^−3^; T is air temperature (°C); $p_{a}$ is atmospheric pressure (P_a_); $p_{m}$ is the added-value of pressure (Pa); $M_{w}$ is relative humidity (%); and $p_{H_{2}O}$ is saturated vapor pressure (Pa);

The procedures of the tests are described below:

Step 1: Measure the leakage volume of the chamber itself $Q_{b}$.

Step 2: Measure the leakage volume of the test specimen and the chamber itself after installing the test specimen $Q_{t}$.

Step 3: Subtract the value obtained from Step 2 from the value obtained from Step 1 to obtain the actual leakage volume of the test specimen $Q_{a}=Q_{t}-Q_{b}$.

Step 4: Correct the value $Q_{a}$ obtained in Step 3 to the leakage volume under standard conditions $Q_{a}’$.

For subsequent discussions, the term “leakage volume” $Q_{a}’$ refers to the leakage volume under standard conditions.

## 3. Results and Discussion

### 3.1. Test Results

**Figure 8:**
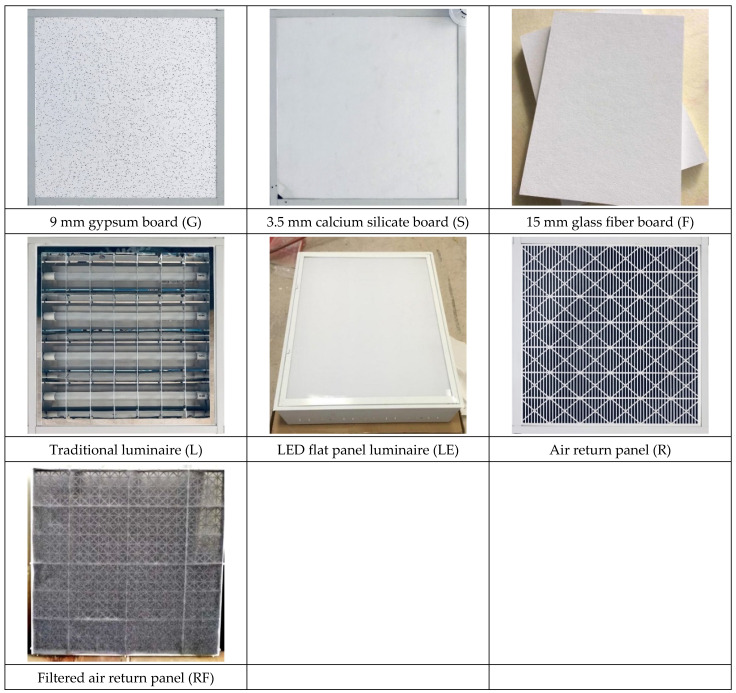
Ceiling specimen numbering diagram.

**Figure 9:**
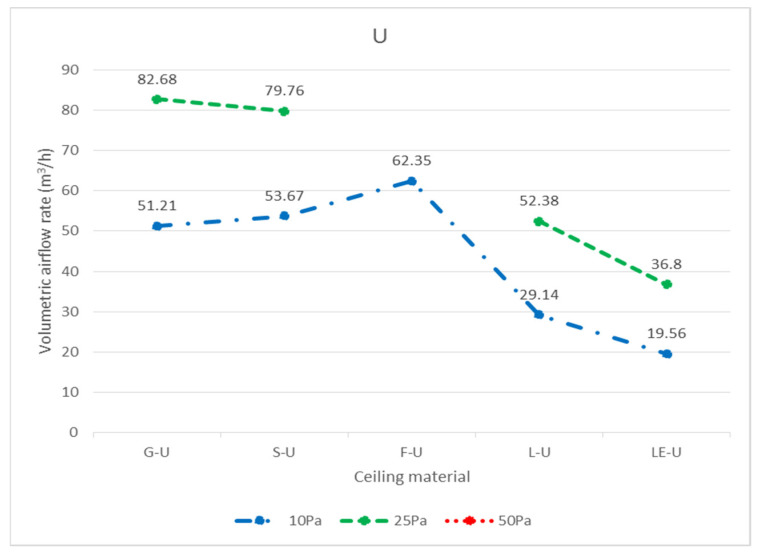
Average leakage volume of each specimen at each pressure difference from bottom to top (U) (m^3^/h).

**Figure 10:**
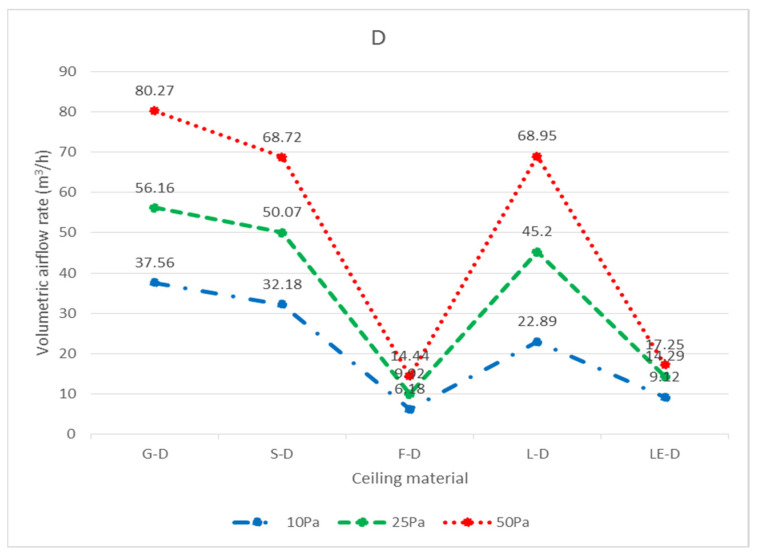
Average leakage volume of each specimen at each pressure difference from top to bottom (D) (m^3^/h).

In total, three widely used ceiling materials were tested: 9 mm gypsum board, 3.5 mm calcium silicate board, and 15 mm glass fiber board, conventional luminaires, LED flat panel luminaires, air return panels, and filtered air return panels for leakage. The test body number of the ceiling is shown in Figure 8; “G” represents 9 mm gypsum board, “S” represents 3.5 mm calcium silicate board, “F” represents 15 mm glass fiber board, and “L” represents traditional luminaire, whereas “LE” represents LED panel luminaire, “R” represents the air return panel, and “RF “ represents filtered air return. The smoke flow direction from top to bottom is designated as D, and the smoke flow direction from bottom to top is designated as U; for the G specimen, G-U-1 represents the leakage amount of 9 mm gypsum board—the smoke flowing from bottom to top—number 1, and G-D-1 represents the leakage amount of 9 mm gypsum board—with the smoke flowing from top to bottom—number 1. G-U-1 was obtained by gluing the other eight specimens (G-U-2–G-U-9) to the gap between the framework with airtight tape, G-U-9 was obtained by gluing the other eight specimens (G-U-1–G-U-8) to the gap between the framework with airtight tape, and so on, to obtain the individual leakage amount of each panel. L, LE, R, and RF specimens were all single devices (of a fixed size) and did not have the same size limitations as the G, S, and F ceiling specimens, for which the uncut die dimensions were designated as No. 5 (these dimensions are used extensively). G, S, and F test specimens No. 1, No. 3, No. 7, and No. 9 had the same dimensions, and the average leakage volume of the four test specimens with the same materials and dimensions can be averaged to obtain the average leakage volume of a test specimen with a dimension of 300 × 300 mm. The average leakage volume of the four test specimens is coded as 33, e.g., labeled as G-U-33 (= ((G-U-1) + (G-U-3) + (G-U-7) + (G-U-9))/4), etc.; No. 2, No. 4, No. 6, and No. 8 are of the same dimensions. By averaging the leakage of four test bodies of the same material and dimensions, the average leakage of 300 × 603 mm can be obtained, with the average leakage of the four test bodies coded as 36, e.g., S-U-36 (= ((G-U-2) + (G-U-4) + (G-U-6) + (G- U-8))/4) U-8))/4), etc. The leakage amount of each specimen is shown in Table 1, which is the average leakage amount after five tests. The value “X” indicates that the leakage volume is too large to measure under corresponding pressure differences. The average leakage volume of each test specimen under each pressure difference is shown in Figure 9 for the case of bottom-up airflow; the average leakage volume of each test specimen under each pressure difference is shown in Figure 10 for the case of top-down airflow.

### 3.2. Leakage Analysis of Specimens

**Figure 11:**
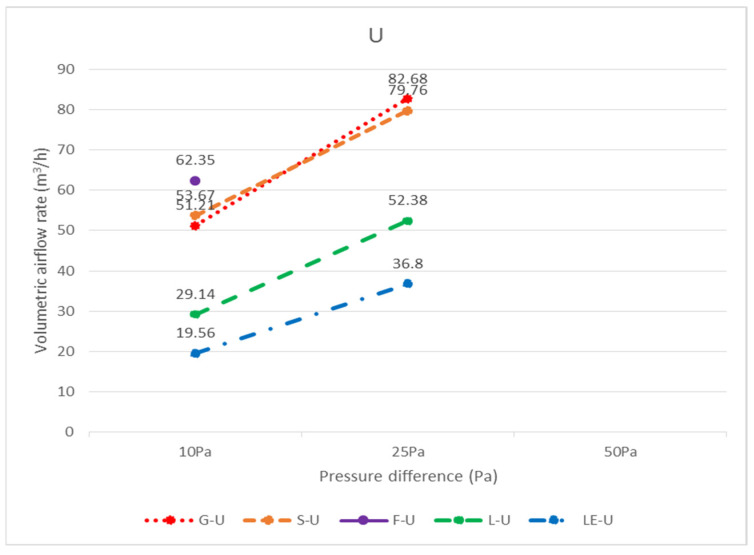
Average leakage of each specimen at each pressure difference from bottom to top (U) (m^3^/h).

**Figure 12:**
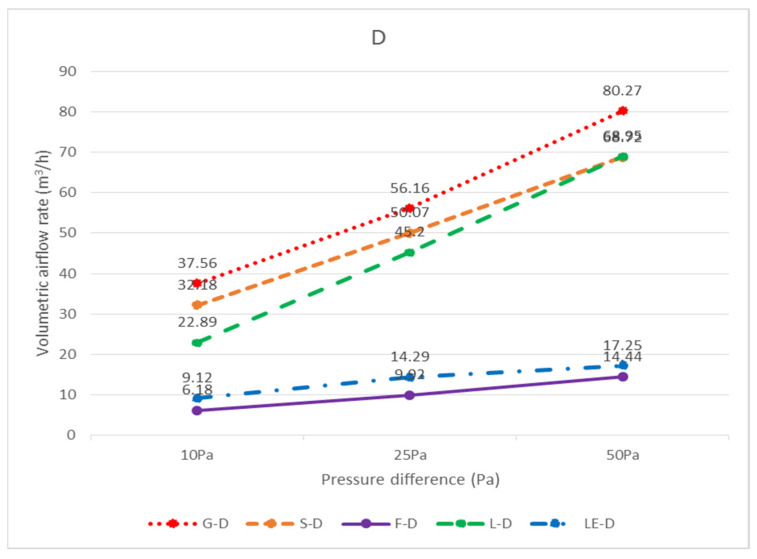
Average leakage of each specimen at each pressure difference from top to bottom (D) (m^3^/h).

The bottom-up leakage of all flow paths was more significant than the top-down leakage of all flow paths. The average leakage of each specimen at each pressure difference for bottom-up flow is shown in Figure 11, and the average leakage of each specimen at each pressure difference for top-down flow is shown in Figure 12. The reason for this is that when the air flows upward, as observed through the transparent acrylic observation port of the chamber, with the pressure starting to exceed the weight of the specimen itself, the specimen will be influenced by the upward buoyancy force and slightly move away from the surface of the light steel frame, resulting in a more significant gap between the specimen and the light steel frame and an increase in the relative leakage volume; on the other hand, when the air flows downward, the surface of the specimen will be subjected to the downward pressure of the airflow and the weight of the specimen itself, resulting in a decrease in the leakage volume between the specimen and the light steel frame. For example, at 10 Pa, G-D-5 (8.54 m^3^/h) is smaller than G-U-5 (19.09 m^3^/h); at 25 Pa, S-D-5 (9.67 m^3^/h) is smaller than S-U-5 (40.58 m^3^/h). At 10 Pa, L-D-5 (22.89 m^3^/h) is smaller than L-U-5 (29.14 m^3^/h), and at 25 Pa, L-D-5 (45.20 m^3^/h) is smaller than L-U-5 (52.38 m^3^/h).

**Figure 13:**
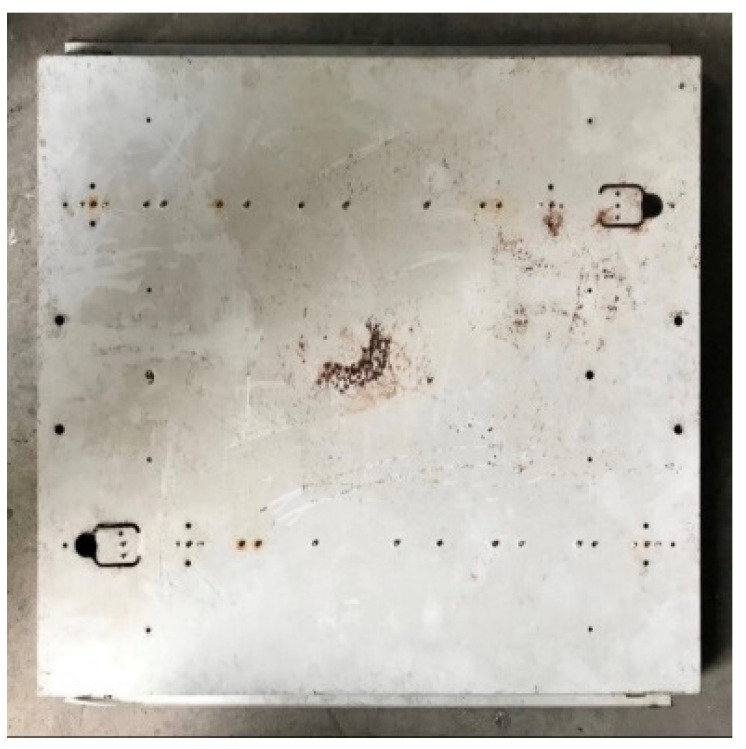
Conventional luminaire (L) with mounting screw holes on the back.

**Figure 14:**
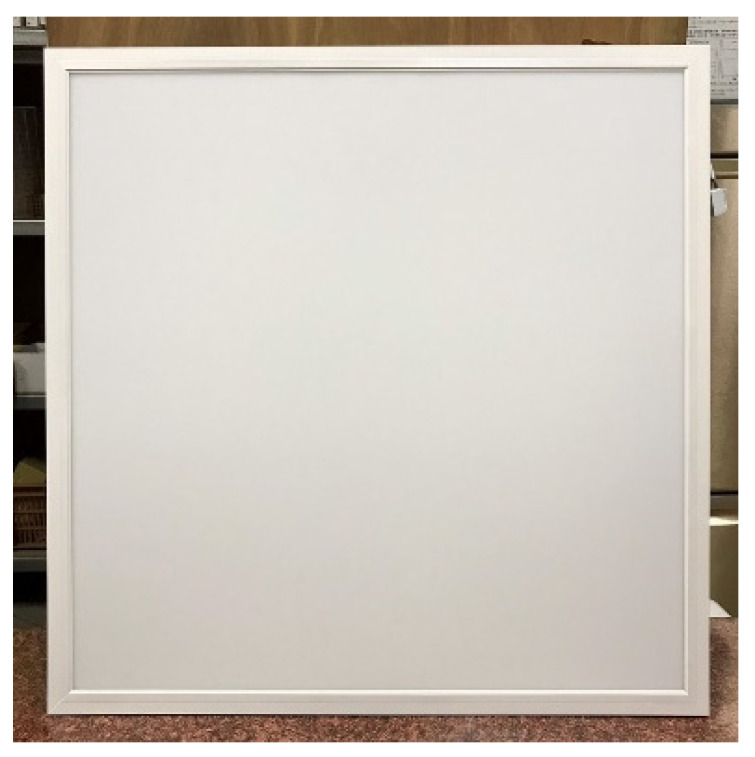
LED flat panel luminaire (LE) without fixing screw holes on the back.

In terms of bottom-up airflow, due to the higher wind pressure at the 50 Pa pressure difference, all the panels, luminaires, and the air return panel itself were not heavy enough to float up and away from the light steel frame, so the corresponding relationship between pressure and leakage volume could not be measured. Altogether, there is a significant relationship between the weight of different specimen densities and the leakage volume. Among the three ceiling materials, the G specimen was the heaviest and was more resistant to floating up as its weight was larger, and thus its relative leakage volume was also smaller. For example, G-U-5 (19.09 m^3^/h) < S-U-5 (20.12 m^3^/h) < F-U-5 (25.12 m^3^/h), G-U-36 (17.05 m^3^/h) < S-U-36 (18.54 m^3^/h) < F-U-36 (20.08 m^3^/h) and G-U-33 (15.07 m^3^/h) < S-U-33 (15.72 m^3^/h) < F-U-33 (17.15 m^3^/h); G-U-5 (35.52 m^3^/h) < S-U-5 (40.58 m^3^/h) < F-U-5 (X m^3^/h), G-U-36 (27.15 m^3^/h) < S-U-36 (31.35 m^3^/h) < F-U-36 (X m^3^/h) and G-U- 33(19.47 m^3^/h) < S-U-33(20.25 m^3^/h) < F-U-33(X m^3^/h). The leakage volume at higher pressure differences was larger than the leakage volume at lower pressure differences, e.g., G-U-5 (35.52 m^3^/h) at 25 Pa > G-U-5 (19.09 m^3^/h) at 10 Pa; S-U-5 (40.58 m^3^/h) at 25 Pa > S-U-5 (40.58 m^3^/h) at 10 Pa. The density of the F specimen was only 0.064 g/cm^3^, and, at 25 Pa differential pressure, the panel was too light and fell off the surface of the framework. As a result, the differential pressure could not be measured; thus, no further leakage test was conducted at 50 Pa. The weight of the LE specimen (2.02 kg) was comparable to that of the G specimen (2.45 kg), with only a difference of 0.43 kg, whereas the thicknesses of the two test specimens were comparable. Both LE and G test bodies had no openings, and their leakage volumes were very similar (LE-U-5 (19.56 m^3^/h) and G-U-5 (19.09 m^3^/h) at 10 Pa; LE-U-5 (36.80 m^3^/h) and G-U-5 (35.52 m^3^/h) at 25 Pa); therefore, it is evident that there is a relationship between weight and leakage volume. The larger the panel area, the greater the upward pressure, and the greater the leakage of the panel, e.g., G-U-5 (19.09 m^3^/h) > G-U-36 (17.05 m^3^/h) > G-U-33 (15.07 m^3^/h), S-U-5 (20.12 m^3^/h) > S-U-36 (18.54 m^3^/h) > S-U-33 (15.72 m^3^/h). 33 (15.72 m^3^/h), F-U-5 (25.12 m^3^/h) > F-U-36 (20.08 m^3^/h) > F-U-33 (17.15 m^3^/h) at 10 Pa; G-U-5 (35.52 m^3^/h) > G-U-36 (27.15 m^3^/h) > G-U-33 (19.47 m^3^/h), S-U-5 (40.58 m^3^/h) > S-U-36 (31.35 m^3^/h) > S-U-33 (20.25 m^3^/h) at 25 Pa. In conclusion, the pressure difference, the weight of the panel, the size of the panel, and the presence of screw holes on the back of the specimen are all related to the leakage volume when the smoke flow is tested in the bottom-up system; the higher the pressure difference, the higher the leakage volume, the higher the weight of the panel, the lower the leakage volume, and the larger the size of the panel, the higher the leakage volume. Therefore, L-U-5 (29.14 m^3^/h) > LE-U-5 (19.56 m^3^/h) at 10 Pa and L-U-5 (52.38 m^3^/h) > LE-U-5 (36.80 m^3^/h) at 25 Pa are also reasonable leakages, as there was a screw hole on the back of specimen L (Figure 13) and no screw hole on the back of specimen LE (Figure 14). During the test, there were too many holes in the R and RF specimens themselves for us to detect the corresponding relationship between pressure difference and leakage volume.

**Figure 15:**
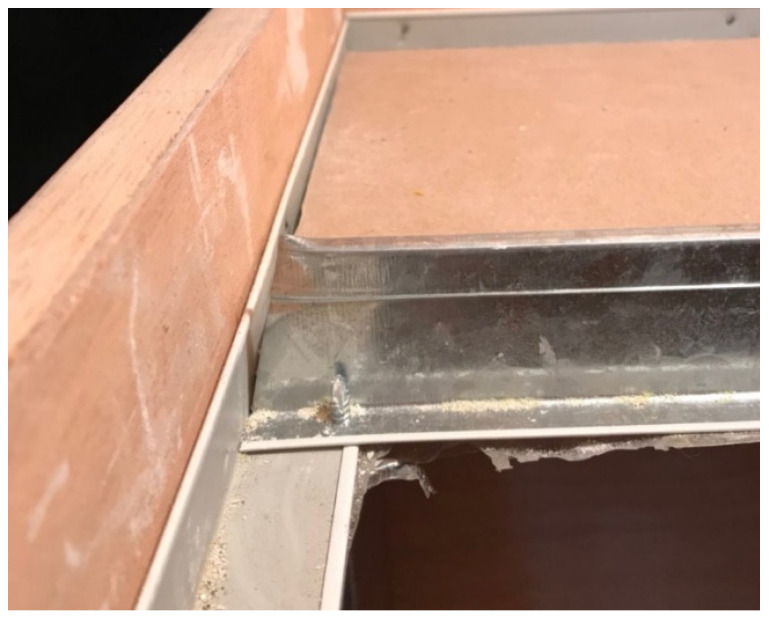
Intersection of frame and trim after cutting.

In terms of top-down airflow, for the three types of plates, the minimum leakage volume was observed for the F specimen, and the maximum leakage volume was observed for the G specimen at 10 Pa, 25 Pa, and 50 Pa pressure differences, e.g., the leakage volume at 10 Pa was F-D-5 (1.72 m^3^/h) < S-D-5 (6.09 m^3^/h) < G-D-5 (8.54 m^3^/h), and the leakage volume at 25 Pa was F-D-5 (2.59 m^3^/h) < S-D-5 (9.67 m^3^/h) < G-D-5 (13.45 m^3^/h). At 25 Pa, the leakage was F-D-5 (2.59 m^3^/h) < S-D-5 (9.67 m^3^/h) < G-D-5 (13.45 m^3^/h), and at 50 Pa, the leakage was F-D-5 (3.51 m^3^/h) < S-D-5 (11.25 m^3^/h) < G-D-5 (16.88 m^3^/h). The larger the pressure difference, the larger the leakage volume, resulting in a positive relationship in the test results; however, theoretically, the same leakage volume should be generated under the same conditions of the same panel area and the same light steel frame when subjected to the same wind pressure from top to bottom. It is understandable that wind pressure from the bottom to the top causes the specimen to move and produce different leakage amounts; yet, when these plates are subjected to the same pressure from top-down, under the same dimensions, and under the same conditions of the light steel frame, they produce different leakage amounts, which would be difficult to notice without conducting actual testing. According to observations made through the transparent acrylic observation port, it was found that the leakage volume was smaller for specimen F because it was easier for the plate and the framework to adhere to each other when subjected to wind pressure, and larger for specimen G because the plate and framework were less likely to adhere to each other when subjected to wind pressure; the amount of leakage was found to be related to the bending load of the specimen itself (36.3 kg/cm^2^ for specimen G, 13.1 kg/cm^2^ for specimen S, and 5.1 kg/cm^2^ for specimen F). The larger the bending loads, the less flexible the panel is, the less likely it is to adhere to the framework when subjected to wind pressure, and the greater the tendency of leakage becomes. For example, at 10 Pa differential pressure, G-D-5 (8.54 m^3^/h) < G-D-36 (11.12 m^3^/h) < G-D-33 (17.90 m^3^/h); at 25 Pa differential pressure, S-D-5 (9.67 m^3^/h) < S-D-36 (17.51 m^3^/h). 36 (17.51 m^3^/h) < S-D-33 (22.89 m^3^/h); and F-D-5 (3.51 m^3^/h) < F-D-36 (4.87 m^3^/h) < F-D-33 (6.06 m^3^/h) at 50 Pa differential pressure. This also overturns the common perception of the general public, whereas the leakage volume of the panel size 300 × 300 mm was the largest and the leakage volume of the panel size 603 × 603 mm was the smallest. There are two main reasons for this result; the first reason is that for the panel size 603 × 603 mm, which is large, the support distance between the two ends of the panel is larger, and the flexibility is also larger, so the gap area is smaller, and the leakage volume is relatively smaller. The second reason is that the size of the 300 × 603 mm panels and 300 × 300 mm panels is smaller, so the distance between the two ends of the panels is smaller, and the flexibility is also smaller, so the gap area is larger, and the leakage volume is also relatively larger. In particular, near the edge of the wall, the light steel frame construction requires the use of hemming material, which is generally cut with scissors (Figure 15), resulting in uneven incisions that are fixed with self-tapping screws at the joints, thus making it impossible for the panels to fully adhere to the surface of the frame, resulting in more gaps and a larger amount of leakage.

### 3.3. Relationship between Specimen Size and Leakage

**Figure 16:**
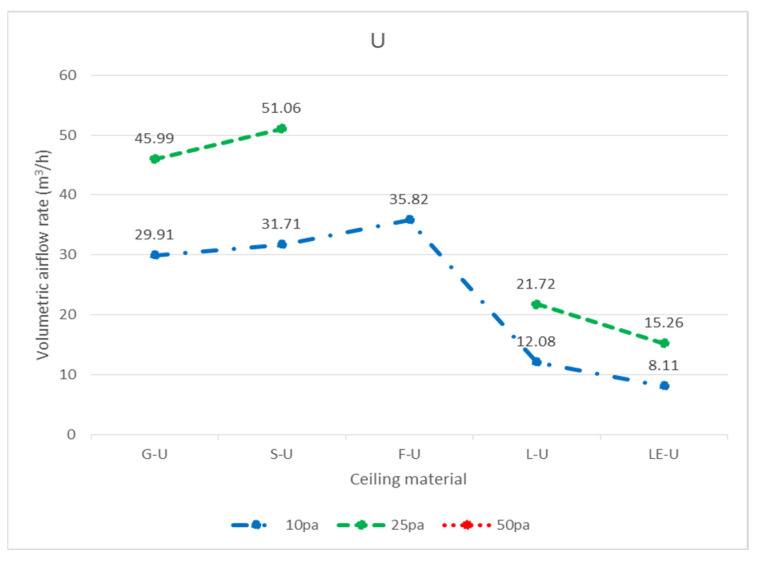
The unit leakage volume (m^3^/(h.m.)) of each specimen under each pressure difference with airflow from bottom to top (U).

**Figure 17:**
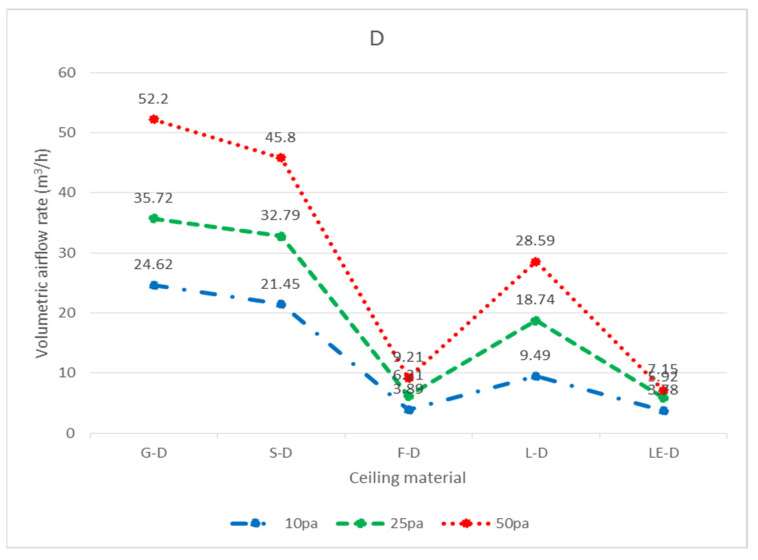
The unit leakage volume (m^3^/(h.m.)) of each specimen under each pressure difference with airflow from top to bottom (D).

**Figure 18:**
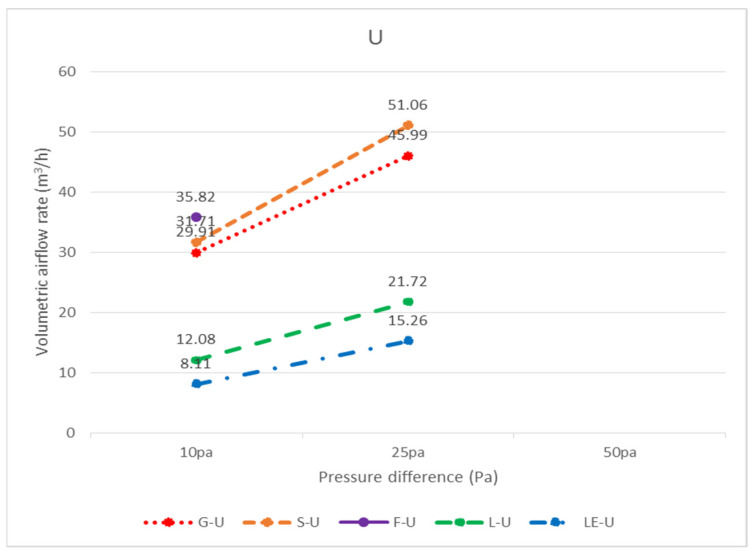
Unit leakage volume (m^3^/(h.m.)) of each specimen at each pressure difference with airflow bottom to top (U).

**Figure 19:**
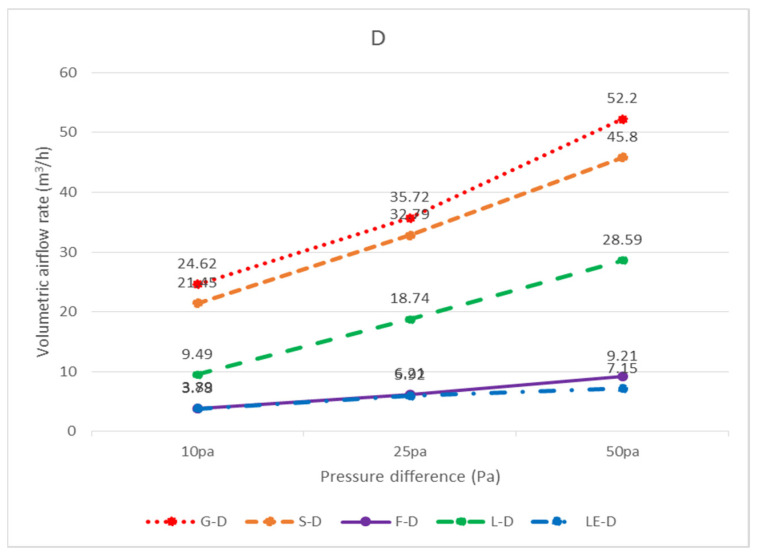
Unit leakage volume (m^3^/(h.m.)) of each specimen at each pressure difference with airflow top to bottom (D).

The leakage amounts of the G, F, S, L, LE, R, and RF specimens with dimensions of 603 × 603 mm can be found in Table 1 in terms of air pressure. However, in general, ceilings are not composed of all integral panels, but rather panels are laid in the middle area as much as possible for the sake of aesthetics, and panels that are laid next to the walls need to be measured and cut on-site; therefore, the dimensions of these so-called closing panels are not fixed, and it is difficult to obtain the leakage amounts of various specimen sizes one by one. Considering the assembly situation of the closing panels, the dimensions of 300 × 603 mm and 300 × 300 mm are more similar; as a result, the analysis of each unit length of 300 × 603 mm and 300 × 300 mm should be sufficient to represent the leakage amount of various closing panels. In the case of top-down air pressure, the leakage volume of G-D-36 was 19.50 m^3^/h with a perimeter of 1.806 m (= 0.3 + 0.603 + 0.3 + 0.603) at 25 Pa, and the leakage volume per unit length was 10.80 m^3^/(h.m); the leakage volume of G-D-33 was 23.21 m^3^/h with a perimeter of 1.2 m (= 0.3 + 0.3 + 0.3 + 0.3), with a leakage rate of 19.34 m^3^/(h.m.) per unit length. The estimated leakage per unit length was different for the two different sized panels; it is recommended that when a panel size is smaller than 300 × 300 mm or between 300 × 300 mm and 300 × 603 mm, different leakage per unit lengths should be incorporated, respectively; if the panel size is larger than 300 × 603 mm and smaller than 603 × 603 mm, the test was not conducted in this study and the leakage per unit length was not estimated. The panels and the light steel frame next to the wall were cut on-site, not uniformly cut in a factory. There are too many variables affecting leakage volume, such as the flatness of the light steel frame itself, the flatness of the intersection of the main steel frame, the sub-frame, and the side steel frame, and the flatness of the panel itself, all of which affect the leakage volume. On-site construction methods, bending of the material specimen, and breaking of the load are directly related to the leakage volume; therefore, it is recommended to adopt a more conservative estimation method for the leakage volume of panels near the wall. The leakage volume of various panel sizes at different pressure differences can also be obtained by following the calculation principle described above for bottom-up air pressure. For top-down air pressure (D), the amount of leakage per unit of length for each specimen size is shown in Table 2; conversely, for bottom-up air pressure (U), the amount of leakage per unit of length for each specimen size is shown in Table 3. The unit leakage amount of each specimen under each pressure difference is shown in Figure 16 for bottom-up airflow and Figure 17 for top-down airflow. The unit leakage of each specimen at each pressure difference for bottom-up airflow is shown in Figure 18, and the unit leakage of each specimen at each pressure difference for top-down airflow is shown in Figure 19.

### 3.4. Evaluation of Evacuation Time

**Figure 20:**
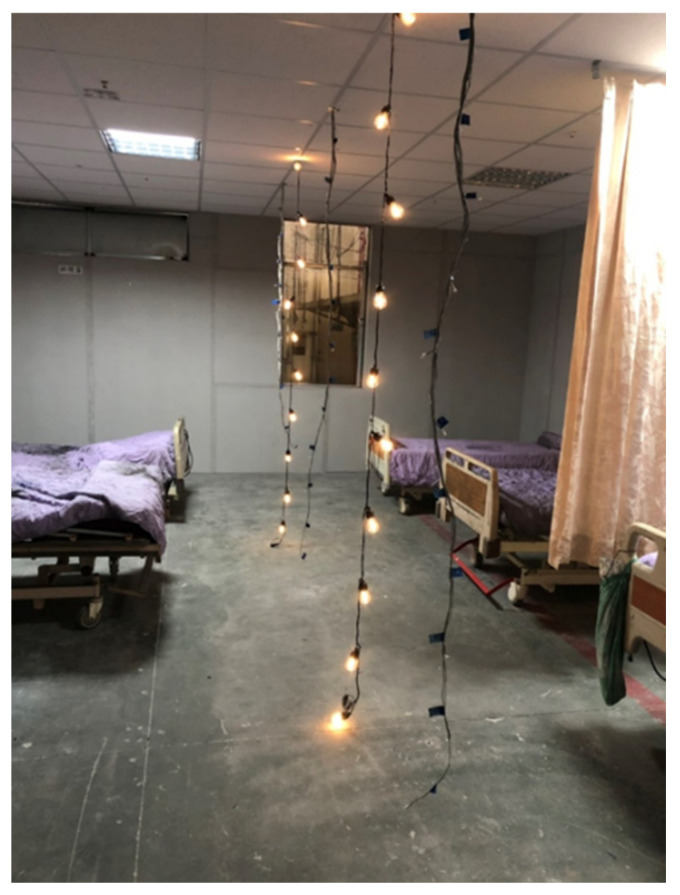
Five-person ward.

To evaluate whether the building occupants can be safely evacuated, time-based evacuation analysis or timed egress analysis is generally adopted across the globe [28], where the actual evacuation time requested safety egress time (REST) must be less than the available safety egress time (ASET) as the benchmark for evaluating the success of the evacuation; the primary reference for the permissible evacuation time is usually the time at which the smoke level drops to 180 cm [29] as the threshold value for evaluation. The evacuation safety theory proposed by Marchant [30] is Tp + Tr + Ta ≦ Tf (Tp: time of fire detection; Tr: time of response to fire; Ta: time of evacuation action; Tf: time when evacuees cannot escape from the environment by themselves). In summary, the time at which the smoke layer drops to a height of 180 cm above the floor is taken as the evacuation permissible time (Tf), which is a critical reference to know when making calculations for evacuations. Thus, by knowing the amount of ceiling leakage and the size of the room, the permissible time for evacuation (Tf) can be calculated. Taking the general nursing home setting standards in Taiwan as an example, two five-person wards form a unit, and the walls of the two wards are only set up to the ceiling, with a net width of 5.9 m, a net depth of 7.9 m, and a floor height of 3 m for each five-person room (the net ceiling height is 2.7 m), with a total of 117 complete panels of ceiling, 13 panels of 50 cm × 60 cm, nine panels of 10 cm × 60 cm. The wards are generally equipped with two T8 lamps with calcium silicate panels, as shown in Figure 20. When the pressure difference is 10 Pa, the leakage of the room in which there is no fire is expressed as follows. The time for the smoke layer to fall to a height of 180 cm above the floor is 5.9 × 7.9 × 0.9/1377.74 × 60 = 1.83 min. The smoke leakage from the fire room upward is 117 × 19.09 + 13 × 17.05 + 9 × 15.07 + 2 × 29.14 = 2649.09 m^3^/h, and the time it takes to fill the space above the ceiling is 5.9 × 7.9 × 0.3 × 2/2649.09 × 60 = 0.63 min. Therefore, when a fire occurs next door, the next ward only has 2.46 min (1.83 + 0.63) of evacuation time. Thus, if we make good use of the results obtained from this study, we can obtain the amount of smoke leakage under each ceiling and each pressure difference and further estimate the smoke descent time to facilitate the calculation of evacuations.

## 4. Conclusions

In the case of bottom-up pressure, the pressure difference, the weight of the panel, the size of the panel, and the presence of screw holes on the back of the specimen are all related to the amount of leakage; the more significant the pressure difference, the more significant the amount of leakage, the larger the weight of the panel, the smaller the amount of leakage, and the larger the size of the panel, the more significant the amount of leakage. The L specimen had screw holes on the back, and the LE specimen had no screw holes on the back; therefore, the amount of leakage was reasonable. During testing, too many holes were found in the R and RF specimens, and the corresponding relationship between the pressure difference and the leakage volume could not be detected.

In the case of top-down pressure, when ceiling dimensions are the same but the ceiling materials are different, the leakage volume will be different, caused by the different bending and load of each panel. The higher the bending loads, the less flexible the panels are, the less likely they are to adhere to the framework when subjected to wind pressure, and the greater the leakage tendency. When the pressure difference was 10 pa, 25 Pa, and 50 Pa, the maximum leakage was from the G plate, and the minimum leakage was from the F plate.

In the case of top-down pressure, when the ceiling material is the same and the ceiling dimensions are different, the maximum leakage was observed for a panel size of 300 × 300 mm, and the minimum leakage was observed for a panel size of 603 × 603 mm. However, when panel dimensions were smaller, the leakage volume tended to be larger, but the leakage volume caused by the largest panel was the smallest. This is because of the use of a large panel size of 603 × 603 mm, which meant that the support distance between the two ends of the panel was larger, and the flexibility was also larger, so the gap area was smaller and the leakage volume was also relatively smaller. This phenomenon is a characteristic of all G panels, S panels, and F panels.

In the case of top-down pressure, panel sizes of 300 × 603 mm and 300 × 300 mm, representing small panel sizes, the support distance between the two ends of the panel was smaller, and the flexibility was also smaller; therefore, the gap area was larger, and the leakage volume was also relatively larger. Furthermore, especially near the edge of the wall, the construction of a light steel frame requires the use of closing materials, which are usually cut with scissors; thus, the cut will not be flat; moreover, the junction area is secured with self-drilling screws; therefore, the panels cannot fully adhere to the surface of the framework, resulting in more gaps and relatively more leaks. This was the case for the G, S, and F panels.

To evaluate the overall unit leakage volume of the ceiling, Table 4 is recommended for the case of top-down pressure (D) and Table 5 for the case of bottom-up pressure (U). When the panel size is smaller than 300 × 300 mm and between 300 × 300 mm and 300 × 603 mm, it is necessary to substitute different extrapolation values to obtain a more accurate leakage value per unit length. When the panel size is 603 × 603 mm, the leakage amount can be applied directly.

Through the application of this study, the smoke leakage value of a room not containing a fire can be evaluated under various pressure differences, which can be used to estimate the smoke descent time to facilitate calculations relating to evacuations. Based on its proven leakage detection capability for ceilings, this apparatus and equipment can be applied to other fire prevention products for smoke detection purposes in the future through the derivation of its system design principles.

## Figures and Tables

**Table 1:** Average leakage volume (m^3^/h) of each specimen at each pressure difference and with different smoke flow directions.

Specimen	Code	Pressure Difference		
		10 Pa	25 Pa	50 Pa
G	G-U-5	19.09	35.52	X
	G-U-36	17.05	27.15	X
	G-U-33	15.07	19.47	X
	Total	51.21	82.68	X
	G-D-5	8.54	13.45	16.88
	G-D-36	11.12	19.50	27.26
	G-D-33	17.90	23.21	36.13
	Total	37.56	56.16	80.27
S	S-U-5	20.12	40.58	X
	S-U-36	18.54	31.35	X
	S-U-33	15.72	20.25	X
	Total	53.67	79.76	X
	S-D-5	6.09	9.67	11.25
	S-D-36	10.09	17.51	24.15
	S-D-33	16.01	22.89	33.32
	Total	32.18	50.07	68.72
F	F-U-5	25.12	X	X
	F-U-36	20.08	X	X
	F-U-33	17.15	X	X
	Total	62.35	X	X
	F-D-5	1.72	2.59	3.51
	F-D-36	1.93	3.44	4.87
	F-D-33	2.53	3.89	6.06
	Total	6.18	9.92	14.44
L	L-U-5	29.14	52.38	X
	L-D-5	22.89	45.20	68.95
LE	LE-U-5	19.56	36.80	X
	LE-D-5	9.12	14.29	17.25
R	R-U-5	X	X	X
	R-D-5	X	X	X
RF	RF-U-5	X	X	X
	RF-D-5	X	X	X

Note: “X” indicates that the leakage volume is too large to measure under the corresponding pressure difference.

**Table 2:** Leakage volume per unit for various test specimen sizes with airflow from top to bottom (D) (m^3^/(h.m)).

Area		603 × 603 (mm)	Below 603 × 603 (mm) Greater than 300 × 300 (mm)	Below 300 × 300 (mm)	Total
Specimen	Pressure Difference				
9 mm gypsum board (G-D)	10 pa	3.54	6.16	14.92	24.62
	25 pa	5.58	10.80	19.34	35.72
	50 pa	7.00	15.09	30.11	52.20
3.5 mm calcium silicate board (S-D)	10 pa	2.52	5.59	13.34	21.45
	25 pa	4.01	9.70	19.08	32.79
	50 pa	4.66	13.37	27.77	45.80
15 mm glass fiber board (F-D)	10 pa	0.71	1.07	2.11	3.89
	25 pa	1.07	1.90	3.24	6.21
	50 pa	1.46	2.70	5.05	9.21
Traditional luminaire (L-D)	10 pa	9.49	Without the size	Without the size	9.49
	25 pa	18.74			18.74
	50 pa	28.59			28.59
LED flat panel luminaire (LE-D)	10 pa	3.78			3.78
	25 pa	5.92			5.92
	50 pa	7.15			7.15
Air return panel (R-D)	10 pa	X			X
	25 pa	X			X
	50 pa	X			X
Filtered air return panel (RF-D)	10 pa	X			X
	25 pa	X			X
	50 pa	X			X

Note: “X” indicates that the leakage volume was too large to measure under the corresponding pressure difference.

**Table 3:** Leakage volume per unit for various test specimen sizes with airflow from bottom to top (U) (m^3^/(h.m.)).

Area		603 × 603 (mm)	Below 603 × 603 (mm) Greater than 300 × 300 (mm)	Below 300 × 300 (mm)	Total
Specimen	Pressure Difference				
9 mm gypsum board (G-U)	10 pa	7.91	9.44	12.56	29.91
	25 pa	14.73	15.03	16.23	45.99
	50 pa	X	X	X	X
3.5 mm calcium silicate board (S-U)	10 pa	8.34	10.27	13.10	31.71
	25 pa	16.82	17.36	16.88	51.06
	50 pa	X	X	X	X
15 mm glass fiber board (F-U)	10 pa	10.41	11.12	14.29	35.82
	25 pa	X	X	X	X
	50 pa	X	X	X	X
Traditional luminaire (L-U)	10 pa	12.08	Without the size	Without the size	12.08
	25 pa	21.72			21.72
	50 pa	X			X
LED flat panel luminaire (LE-U)	10 pa	8.11			8.11
	25 pa	15.26			15.26
	50 pa	X			X
Air return panel (R-U)	10 pa	X			X
	25 pa	X			X
	50 pa	X			X
Filtered air return panel (RF-U)	10 pa	X			X
	25 pa	X			X
	50 pa	X			X

Note: “X” indicates that the leakage volume was too large to measure under the corresponding pressure difference.

**Table 4:** Leakage volume per unit for various test specimen sizes from top to bottom (D) (m^3^/(h.m)).

**Panel Size (mm)**	**Pressure Differences of Panel Types**								
	**9 mm Gypsum Board (G-D)**			**3.5 mm Calcium Silicate Board (S-D)**			**15 mm Glass Fiber Board (F-D)**		
	**10 Pa**	**25 Pa**	**50 Pa**	**10 Pa**	**25 Pa**	**50 Pa**	**10 Pa**	**25 Pa**	**50 Pa**
Each piece 603 × 603 (mm)	3.54	5.58	7.00	2.52	4.01	4.66	0.71	1.07	1.46
300 × 603–603 × 603 (mm)	6.16	10.80	15.09	5.59	9.70	13.37	1.07	1.90	2.70
Below 300 × 300 (mm)	14.92	19.34	30.11	13.34	19.08	27.77	2.11	3.24	5.05
Total	24.62	35.72	52.20	21.45	32.79	45.80	3.89	6.21	9.21
**Panel Size (mm)**	**Pressure Differences of Panel Types**								
	**Traditional Luminaire (L-D)**			**LED Flat Panel Luminaire (LE-D)**			**Air return Panel (R-D), Filtered Air Return Panel (RF-D)**		
	**10 Pa**	**25 Pa**	**50 Pa**	**10 Pa**	**25 Pa**	**50 Pa**	**10 Pa**	**25 Pa**	**50 Pa**
Each piece 603 × 603 (mm)	9.49	18.74	28.59	3.78	5.92	7.15	X	X	X
300 × 603–603 × 603 (mm)	Without the size								
Below 300 × 300 (mm)	Without the size								
Total	9.49	18.74	28.59	3.78	5.92	7.15	X	X	X

Note: “X” indicates that the leakage volume was too large to measure under the corresponding pressure difference.

**Table 5:** Leakage volume per unit for various test specimen sizes from bottom to top (U) (m^3^/(h.m.)).

**Panel Size (mm)**	**Pressure Differences of Panel Types**								
	**9 mm Gypsum Board (G-U)**			**3.5 mm Calcium Silicate Board (S-U)**			**15 mm Glass Fiber Board (F-U)**		
	**10 Pa**	**25 Pa**	**50 Pa**	**10 Pa**	**25 Pa**	**50 Pa**	**10 Pa**	**25 Pa**	**50 Pa**
Each piece 603 × 603 (mm)	7.91	14.73	X	8.34	16.82	X	10.41	X	X
300 × 603–603 × 603 (mm)	9.44	15.03	X	10.27	17.36	X	11.12	X	X
Below 300 × 300 (mm)	12.56	16.23	X	13.10	16.88	X	14.29	X	X
Total	29.91	45.99	X	31.71	51.06	X	35.82	X	X
**Panel Size (mm)**	**Pressure Differences of Panel Types**								
	**Traditional Luminaire (L-U)**			**LED Flat Panel Luminaire (LE-U)**			**Air return Panel (R-U),** **Filtered Air Return Panel (RF-U)**		
	**10 Pa**	**25 Pa**	**50 Pa**	**10 Pa**	**25 Pa**	**50 Pa**	**10 Pa**	**25 Pa**	**50 Pa**
Each piece 603 × 603 (mm)	12.08	21.72	X	8.11	15.26	X	X	X	X
300 × 603–603 × 603 (mm)	Without the size								
Below 300 × 300 (mm)	Without the size								
Total	12.08	21.72	X	8.11	15.26	X	X	X	X

Note: “X” indicates that the leakage volume was too large to measure under the corresponding pressure difference.

## Data Availability

Data is contained within the article. The data presented in this study are available in Table 4 and Table 5.
